# Ti Ions Induce IL-1β Release by Activation of the NLRP3 Inflammasome in a Human Macrophage Cell Line

**DOI:** 10.1007/s10753-022-01672-7

**Published:** 2022-06-21

**Authors:** Mattias Pettersson, Sanna Almlin, Georgios E. Romanos, Anders Johansson

**Affiliations:** 1 Division of Prosthetic Dentistry, Department of Odontology, Faculty of Medicine, Umeå University, SE-901 87, Umeå, Sweden; 2 Molecular Periodontology, Department of Odontology, Faculty of Medicine, Umeå University, Umeå, Sweden; 3 Department of Periodontology, School of Dental Medicine, Stony Brook University, Stony Brook, NY 11794 USA

**Keywords:** NLRP3 inflammasome, Titanium, Interleukin-1β, Macrophage cell line

## Abstract

The aim of the present study was to investigate whether titanium (Ti)-induced release of interleukin (IL)-1β acts through the assembly of the NACHT, LRR, and PYD domain-containing protein 3 (NLRP3) inflammasome. In addition, we examined whether particulate Ti or TiO_2_ activates the same intracellular pathways with the assembly of the NLRP3 inflammasome as Ti ions. Ti ions are known to induce IL-1β maturation and release by the formation of metal–protein aggregates. Wild-type THP-1 (wt.) cells and NLRP3^−^ and ASC^−^ (apoptosis-associated speck-like protein containing caspase recruitment domain (CARD)) knockdown cells were used in the experimental analyses. Macro- and nanoparticles (NPs) of both Ti and TiO_2_ were used as test agents. IL-1β release as a biomarker for inflammasome activation and cell viability was also analyzed. Periodate-oxidized adenosine triphosphate (oATP) was used to attenuate downstream signaling in NLRP3 inflammasome activation. Cellular uptake of Ti was examined using transmission electron microscopy. Cells exposed to the Ti-ion solution showed a dose-dependent increase in the release of IL-1β; conversely, exposure to particulate Ti did not result in increased IL-1β release. Cell viability was not affected by particulate Ti. Knockdown cells exposed to Ti showed a statistically significant reduction in the release of IL-1β compared with wt. cells (*p* < 0.001). Cellular uptake was detected in all Ti mixtures, and aggregates with various structures were observed. Ti ion–induced release of bioactive IL-1β in THP-1 cells involves the assembly of the NLRP3 inflammasome.

## Background

Titanium dioxide (TiO_2_) is one of the most commonly utilized nanoparticles (NPs). Environmental exposure to these particles, and the consequent safety concerns, is under scrutiny because of their wide range of applications in industry, personal care, and consumer products (e.g., paint, food, dental, implants, medical devices, and cosmetics) [1]. Titanium (Ti) is known to induce inflammation by releasing the pro-inflammatory cytokine interleukin (IL)-1β, thereby causing inflammatory pyroptotic cell death [2–4]. IL-1β is regulated by the cytosolic multiprotein oligomer, NLRP3 inflammasome, comprising a sensor (NLRP3), an adaptor (ASC), and an effector (caspase-1) [5]. The induction and release of bioactive IL-1β are triggered by the activation of toll-like receptors through recognition of pathogen-associated molecular patterns (PAMPs), tumor necrosis factor receptor (TNFR), or IL-1 receptor type 1 (IL-1R1), leading to the release of nuclear factor-κβ (NF-κβ) into the cytosol. The transcription of the NF-κβ signaling gene is initialized in the cell nucleus, leading to the upregulation of pro-IL-1β production [6]. The NLRP3 inflammasome regulates caspase-1, a protein that cleaves pro-IL-1β into bioactive IL-1β. Regulation of the oligomerization of NLRP3 inflammasome components is triggered by a secondary signal (e.g., RNA virus, pore-forming toxins, ATP, particulates, and crystals) [5].

It is believed that Ti ions function as a secondary intra cellular danger signal in the assembly of the components of the NLRP3 inflammasome [2]. TiO2 NPs are also known to activate the NLRP3 inflammasome in macrophages, inducing inflammation, which causes diseases similar to silicosis and asbestosis in the lung tissue [7–9]. Ti aggregates have been shown to be phagocytized and located in the lysosomes of exposed macrophages [10]. It is likely that Ti particles are degraded by lysosomal products such as reactive oxygen species (ROS) in the phagolysosome. Due to the inherent stability of these particles, the lysosomal membrane ruptures and cysteine proteinase cathepsin B leaks into the cytosol [11]. This proteinase acts as a damage-associated molecular pattern (DAMP) interacting with NLRP3 at the endoplasmic reticulum level, initiating oligomerization of the NLRP3 inflammasome [11, 12].

It has been shown that lysosomes play a key role in both priming and assembly of the NLRP3 inflammasome, resulting in release of bioactive IL-1β [13]. NLRP3 inflammasome activation in human monocytes can be induced through the activation of the P2X_7_ receptor by the release of endogenous ATP, which occurs after exposure to lipopolysaccharides (LPS) [14]. Periodate-oxidized adenosine triphosphate (oATP) is a known antagonist of the P2X receptor that attenuates the pro-inflammatory response [15]. The IL-1 family of cytokines is central mediators of innate immunity and inflammation [16].

It is currently known that Ti can aggravate the immunological response in the peri-implant tissue and possibly contribute to the rapid destruction of supporting tissue around dental implants [2, 4, 17–19]. Ti debris begins to accumulate in the oral tissue right from the time of dental implant installation [20]. Further release of Ti particles around dental implants can be a direct consequence of acids produced by bacteria, mechanical stress (mastication), or a combination of the two [21]. Peri-implantitis treatment often includes mechanical removal of the biofilm. It has been shown that ultrasonic scaling around dental implants leads to the release of Ti microparticles *in vitro* [22]. It has been shown that patients with peri-implantitis have a higher Ti level in the submucosal plaque around dental implants than patients with healthy dental implants [23]. To our knowledge, no previous studies have investigated if different forms and particle sizes of Ti activates the NLRP3 inflammasome in human macrophages. The hypothesis is that phagocytized Ti induces disruption of the lysosome membrane and consequently release of molecules with capacity to activate IL-1β released through activation of the NLRP3 inflammasome.

In the present study, we investigated whether the Ti-induced release of IL-1β acts through the assembly of the NLRP3 inflammasome. Second, we investigated whether particulate Ti and TiO_2_ activate a similar intra cellular process, as Ti ions in a human macrophage cell line.

## Materials and Methods

### Growth Medium and Cell Culture

A human acute monocytic leukemia cell line THP-1 (ATCC® TIB-202TM) obtained from the American Type Culture Collection (ATCC®; Manassas, VA, USA), cultured in RPMI 1640 containing 10% fetal bovine serum (FBS) supplemented with penicillin–streptomycin (Sigma-Aldrich; St Louis, MO, USA), was used herein. Wild-type THP-1 cells (wt.) and cells with inflammasome components knocked down, namely NLRP3^−^ and ASC^−^ (InvivoGen, Toulouse, France), were used for the various experimental procedures. THP-1 -ASC^−^ and NLRP3^−^ cells were generated from the human monocytic THP1-Null2 cell line through knockdown of the ASC or NLRP3 gene, causing the reduction of ASC or NLRP3 expression. Activation of caspase-1 is substantial downregulated in NLRP3^−^ and ASC^−^ cells. In the experiments evaluating the uptake of soluble and particulate Ti, only THP-1 wt. cells were used.

### Stimulation Agents

A plasma standard solution Specpure®, 1000 µg/mL Ti, from Alfa Aesar (Haverhill, MA, USA) was used as the ion solution for Ti. The metal was stabilized in an ionic form by the acid content in the plasma standards, 5% HNO_3_/trace (tr) hydrogen fluoride (HF). Acidity was determined as previously described [2]. Macro particles: Ti (99%, 5 µm); TiO_2_ (rutile, 1.5 µm) and nanoparticles: Ti (99.9 + %, 30–50 nm, metal basis); TiO_2_ (rutile, high purity, 99.9 + %, 30 nm) from US Research Nanomaterials, Inc. (Houston, TX, USA) were used.

### Cell Stimulation

One-hundred microliters of THP-1 cells (wt., NLRP3^−^, or ASC^−^) suspended in RPMI 1640 was seeded in a 96-well culture plate at a cell concentration of 10^6^ cells/mL. Phorbol 12-myristate 13-acetate (PMA) (Sigma-Aldrich) was added at a concentration of 50 nM, and the cells were incubated at 37 °C under 5% CO_2_ for 24 h to differentiate THP-1 cells toward the macrophage phenotype. The PMA–stimulated THP-1 cells changed from a planktonic to an adherent phenotype with increased sensitivity to inflammasome stimulation [24].

After 24 h of culture at 37 °C under 5% CO_2_, THP-1 cells were primed with 100 ng/mL LPS from *Escherichia coli* (Sigma-Aldrich) for 6 h. Thereafter, the growth medium was replaced with 100 mL of RPMI 1640 containing stimulatory agents, Ti ions, particulate Ti (macro- or nanoparticles), or particulate TiO_2_ (macro- or nanoparticles) at a concentration range of 0–500 µM. The cells were exposed to different agents for 18 h, after which the experiment was terminated. In the experiments with knockdown cells, only Ti-ion solution was used as the stimulation agent in the concentration range of 0–1000 µM.

Only wt. cells were used in the experiments in which oATP (Merck KGaA, Darmstadt, Germany) was added. oATP, at a concentration of 500 µM, was added to the cells before exposure to the Ti-ion solution at a concentration range of 0–900 µM. The experiment was terminated after 18 h of exposure to the test solution.

### Cytokine Analyses with ELISA

The release of bioactive IL-1β from the cells was used as a biomarker for the activation of the NLRP3 inflammasome. Quantification of secreted bioactive IL-1β in the supernatant was performed by ELISA (Human IL- 1β DuoSet ELISA; R&D Systems, Inc. Minneapolis, MN, USA). After 18 h of exposure of the cells, the supernatant was removed, and ELISA was performed following the manufacturer’s protocol. A spectrophotometer (Multiskan Go, Thermo Fisher Scientific Inc., Waltham, MA, USA) at a wavelength of 450 nm was used to quantify the amount of IL-1β secreted into the supernatant.

### Cytotoxicity

Cell viability was analyzed using the neutral (3-amino-7-dimethylamino-2-methylphenazine hydrochloride) red uptake assay (NRU), following a standard protocol [25]. The viability and absorbance of the extracted neutral red were measured with Multiskan Go at a wavelength of 540 nm, using the blanks without cells as the reference. The neutral red uptake in the control cells cultured in plain medium was considered 100% viable cells.

### Cell Uptake Investigated with Transmission Electron Microscopy (TEM)

The Ti ion, particulate Ti (5 µM; 5 µm or 30–50 nm), and TiO_2_ (1.5 µm or 30 nm) solutions were prepared by suspending in RPMI 1640 ± 10% FBS up to a concentration of 200 µM. THP-1 cells (wt%) were exposed to different stimulation agents for 18 h, following which the experiment was terminated. THP-1 cells were fixed using 2.5% glutaraldehyde in 0.1 M sodium cacodylate buffer, washed, and scratched off from the culture plate. Cell suspensions were post-fixed in 1% osmium tetroxide, dehydrated with ethanol and propylene oxide, and finally embedded in Spurr resin (TAAB, Aldermaston, Berks, England), according to standard procedures [26]. After fixation, the cells were sectioned with an ultramicrotome into 80-nm thick sections. Sections were contrasted with uranyl acetate and lead citrate and examined with a Talos 120C (FEI, Eindhoven, The Netherlands) operating at 120 kV. Micrographs of the cells were acquired with a Ceta 16 M CCD camera (FEI, Eindhoven, Netherlands) using TEM Image and Analysis software ver. 4.14 (FEI). The open-source program FIJI (FIJI is just ImageJ) (https://fiji.sc/) was used for post-processing images of Ti cell uptake, acquired with TEM [27].

### Statistical Analysis

Variance analysis of released IL-1β from wt., NLRP3^−^, and ASC^−^ cells was calculated with one-way ANOVA, using the Sidak correction test for multiple comparisons between groups. ANOVA was also used for variance analyses of IL-1β released from THP-1 cells exposed to particulate Ti and Ti-ion solutions. Data were considered significant at *p* ≤ 0.05. Micrographs of the cells acquired by TEM are described in detail. Statistical analyses were performed with Prism version 9.3.1 (GraphPad Software, San Diego, CA, USA).

## Results

### IL-1β Release and Viability in wt., NLRP3^−^, and ASC^−^ cells

**Fig. 1:**
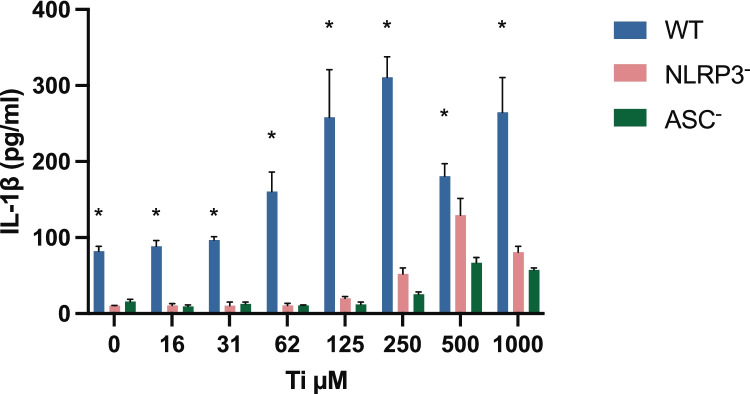
Effect of Ti on IL-1β release from PMA–differentiated THP-1 cells, wt., NLRP3^−^, and ASC^−^. The cells were exposed to Ti for 18 h and the amount of IL-1β released into the culture supernatant analyzed at the end of the exposure period. Mean ± SD of triplicates is shown. Wt. cells show a significantly higher release of IL-1β at all concentrations, at a significance level of *p* < 0.05. *Ti* titanium, *PMA* phorbol 12-myristate 13-acetate, *wt.* wild-type, *NLRP3* NACHT, LRR, and PYD domain-containing protein 3, *ASC* apoptosis-associated speck-like protein containing a CARD.

**Fig. 2:**
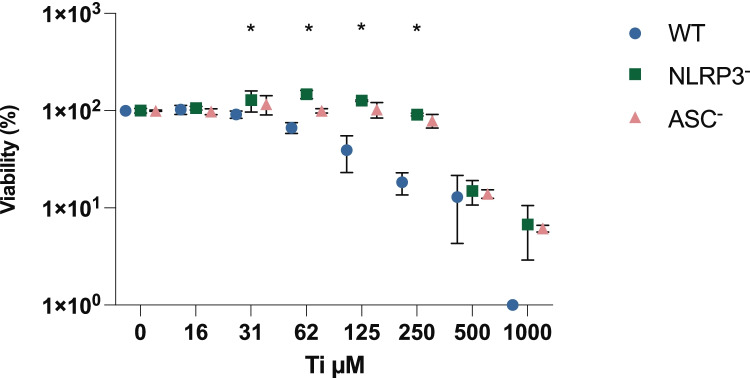
Effect of Ti on the viability of PMA–differentiated THP-1 cells, wt., NLRP3^−^, and ASC^−^. The cells were exposed to Ti for 18 h and the viability analyzed at the end of the exposure period. Mean of triplicates is shown as percent of the control cells not exposed to Ti. Viability of the wt. cells decreased significantly compared with the viability of the knockdown cells in the concentration range 31–250 µM, at a significance level of *p* < 0.05. *Ti* titanium, *PMA* phorbol 12-myristate 13-acetate, *wt.* wild-type, *NLRP3* NACHT, LRR, and PYD domain-containing protein 3, *ASC* apoptosis-associated speck-like protein containing a CARD.

THP-1 cells (wt%) exposed to Ti showed a dose-dependent increase in the release of bioactive IL-1β, reaching a maximum at a concentration of 250 µM. Both NLRP3^−^ and ASC^−^ cell types showed a statistically significant (*p* < 0.001) lower release of active IL-1β compared with active IL-1β release seen in wt. cells (Fig. 1). The viability of wt. cells exposed to Ti began to decrease at 62 µM, with total cell death observed at 1000 µM (Fig. 2). Both cell types with the inflammasome component knocked out showed a statistically significant (*p* < 0.001) higher viability in the interwall concentration tested (Fig. 2). Control cells primed with *E. coli* LPS also showed a significantly higher release of IL-1β in the wt. cells than in the knockdown cells.

### Effect on IL-1β Release in the Presence of Periodate-Oxidized Adenosine Triphosphate (oATP)

**Fig. 3:**
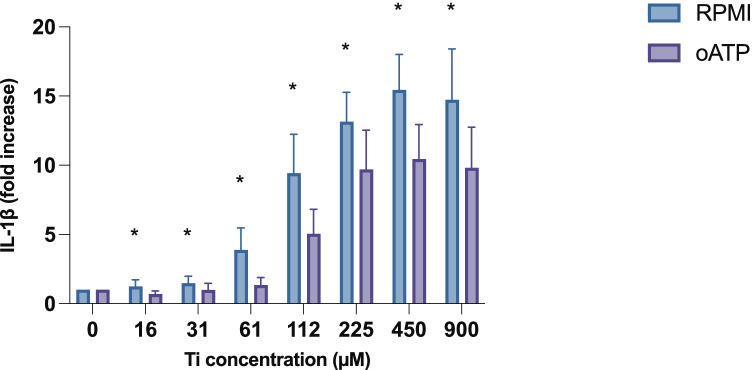
Effect of Ti on IL-1β release from PMA–differentiated THP-1 cells in RPMI 10% FBS ± oATP. Mean ± SD of triplicates is shown. Cells subjected to oATP treatment show a significantly lower rate of IL-1β release at all concentrations, at a significance level of *p* < 0.05. *Ti* titanium, *PMA* phorbol 12-myristate 13-acetate, *FBS* fetal bovine serum, *oATP* periodate-oxidized adenosine triphosphate.

**Fig. 4:**
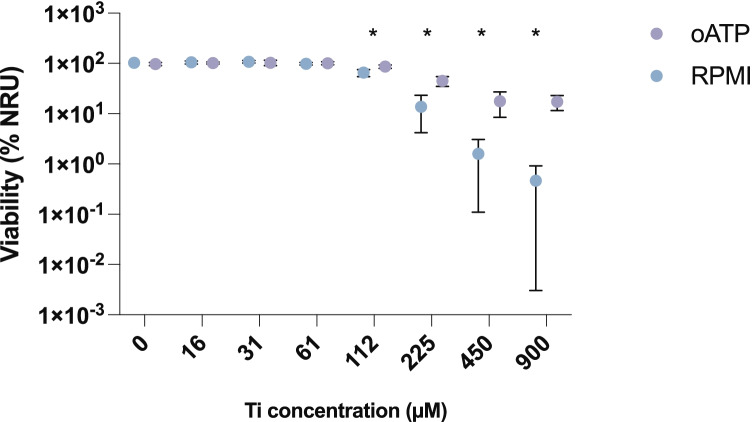
Effect of Ti on the viability of PMA–differentiated THP-1 cells in RPMI 10% FBS ± oATP. Mean ± SD of triplicates is shown. Cell viability was lower among cells not treated with oATP in the concentration range 112–900 µM, at a significance level of *p* < 0.05. *Ti* titanium, *PMA* phorbol 12-myristate 13-acetate, *FBS* fetal bovine serum, *oATP* periodate-oxidized adenosine triphosphate.

A dose-dependent increase in the release of IL-1β was observed at the tested concentrations of 0–900 µM, reaching a maximum at 450 µM (Fig. 3). In the presence of oATP, a significantly lower release of IL-1β was observed at all concentrations in the range 16–900 µM (*p* < 0.05). The Ti solution had no effect on cell viability at concentrations of up to 61 µM, and the viability began to decrease. Cells with oATP added to the supernatant showed significantly higher cell viability than cells without the additive (*p* < 0.001) (Fig. 4). Total cell death occurred at a Ti concentration of 450 µM, whereas cells with added oATP did not reach total cell death at the tested concentrations.

### IL-1β Release and Viability in Cells Exposed to Ti-Ion Solution, Ti- or TiO_2_-Particles

**Fig. 5:**
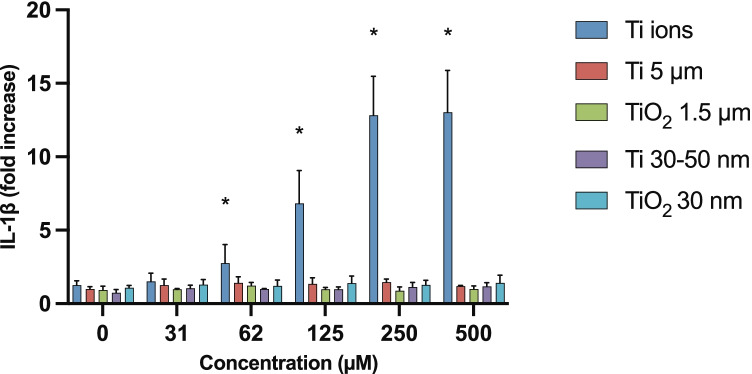
Effect on IL-1β release from PMA–differentiated THP-1 wt. cells after exposure to Ti ions, particulate Ti, or TiO_2_ (macro or nano particles) for 18 h. Mean ± SD of duplicates is shown. Cells exposed to Ti ions show a statistically significantly higher rate of IL-1β release in the concentration range 62–500 µM compared with cells exposed to the different forms of particulate Ti or TiO_2_ at a significance level of *p* < 0.05. *PMA* phorbol 12-myristate 13-acetate, *Ti* titanium.

**Fig. 6:**
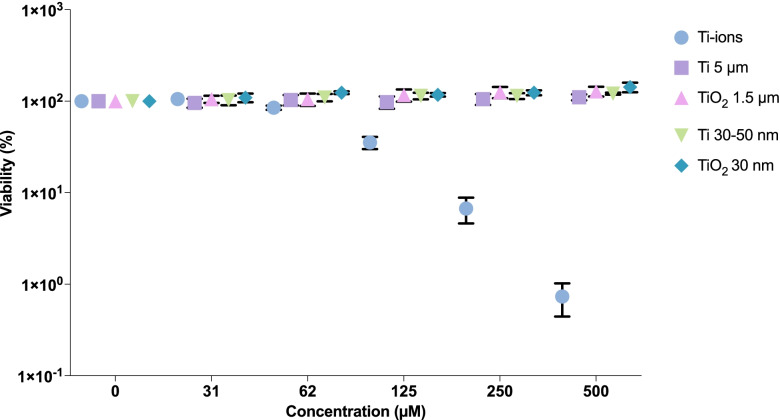
Effect of Ti on the viability of PMA–differentiated THP-1 cells exposed to Ti ions, macro- or NPs of Ti, or TiO_2_. Mean of duplicates is shown as percent of the control cells not exposed to Ti. Viability of cells exposed to Ti ions decreased significantly compared with the viability of cells exposed to the different forms of particulate Ti or TiO_2_. Additionally, TiO_2_ NPs showed a statistically significantly increase in the viability of the cells compared with the viability seen in the control at a concentration of 500 µM. Significance level was *p* < 0.05. *Ti* titanium, *PMA* phorbol 12-myristate 13-acetate, *NP* nanoparticle.

Cells exposed to Ti-ion solution showed a dose-dependent increase in the release of IL-1β into the supernatant (Fig. 4). Cells exposed to either Ti or TiO_2_ (macro- or NPs) did not show any increase in the release of IL-1β in the tested concentration range during an exposure time of 18 h (Fig. 4). The viability of the cells exposed to the different forms of particulate Ti or TiO_2_ showed a slight increase (Fig. 5). At a concentration of 500 µM, TiO_2_ showed a statistically significant increase in viability compared with the control (*p* < 0.001). Cells exposed to Ti ions showed a significant decrease in viability compared with cells exposed to the particulate forms of Ti and TiO_2_ in the concentration range of 62–500 µM, with total cell death at 500 µM (*p* < 0.001) (Fig. 6).

**Fig. 7:**
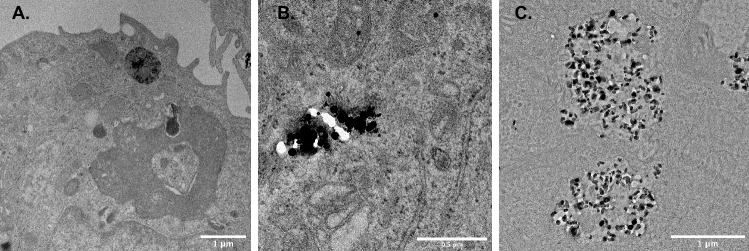
Transmission electron microscope image shows THP-1 cells with uptake of **A** Ti–protein aggregates inside an organelle, with a clear visible membrane structure, as previously shown [10]; **B** Ti NPs; and **C** TiO_2_ NPs inside the cytosol without any clear membrane structure seen encapsulating the particles. *Ti* titanium, *NP* nanoparticle.

### Transmission Electron Microscopy Investigation of Macrophages Exposed to Soluble or Particulate Ti or TiO_2_

TEM investigation of cells exposed to different stimulation agents showed the presence of Ti or TiO_2_ inside all the cells investigated. In the cells exposed to Ti ions, Ti could be identified inside the cell, with a visible membrane structure surrounding the Ti (Fig. 7a); furthermore, in cells exposed to NPs of Ti and TiO_2_, the particles could be identified using TEM. These particles were observed in the cytosol of the cells, with no clear visible membrane structure surrounding them (Fig. 7b, c). In addition, Ti and TiO_2_ could be identified using TEM inside the cytosol of the cells exposed to macroparticles, showing the same pattern as NPs (data not shown). The images acquired during the TEM investigation showed a clear difference in the appearance of the Ti NPs and TiO_2_. The Ti NPs were seen as dark circular particles inside the cells, whereas TiO_2_ was seen as crystals, with a greater variety in geometric forms and radiolucency.

## Discussion

The primary objective of the present study was to investigate whether Ti-induced IL-1β release works through activation of the NLRP3 inflammasome in human macrophages. In the absence of the inflammasome components ASC and NLRP3 in knockdown cells used in the experiments, the release of bioactive IL-1β was significantly reduced. This shows that the pro-inflammatory capability of Ti ions works through the assembly of the NLRP3 inflammasome, which confirms our findings in a previous study [2]. It is known that cell death by pyroptosis is induced in phagocytes that engulf stable particles incapable of being decomposed in the phagolysosome, which in turn induces the assembly of the inflammasome complex [28, 29].

The viability of cells exposed to Ti ions was augmented in ASC^−^ and NLRP3^−^ cells. This indicates that cell death caused by Ti ions is due to activation of the NLRP3 inflammasome, which in turn leads to cell death by pyroptosis. Assembly of the inflammasome will not occur in the absence of inflammasome components in the knockdown cells, which also reduces endogenous-induced cell death. These findings were strengthened by the addition of oATP to the cells, which led to increased viability. Blocking P2X_7_ receptors with oATP inhibits the pro-inflammatory response and also blocks the induction of pyroptosis [14, 30]. Our findings obtained herein showed that the viability of cells exposed to Ti ions increased in the presence of oATP, suggesting that inflammasome activation plays a crucial role in the cytotoxicity of Ti ions in human macrophages by blocking the ability of cells to induce cell death by pyroptosis.

In the tested concentration range of 0–500 µM, neither macro- nor nanoparticles of Ti or TiO_2_ induced IL-1β release from the tested cells, whereas Ti ions showed a dose-dependent increase in release. Our present findings are in contrast to findings in other studies, where TiO_2_ NPs showed an effect on the release of active IL-1β and viability [31]. In that study, NPs with a diameter of 21 or 35 nm were employed; smaller NPs induced cell death and larger NPs induced an inflammatory response. The smaller NPs induced cell mitochondrial dysfunction, which in turn led to cell death. The 35-nm TiO_2_ NPs induced an inflammatory response after autophagy of the particles used in the experiments. In the present study, we used TiO_2_, with a size of 30 nm, and Ti NPs, with a size of 30–50 nm. Additionally, a human cell line was used instead of the mouse cell line used by Hu et al. [31], which explains the distinctive findings. Numerous studies have shown pro-inflammatory and cytotoxic effects of exposing macrophages to Ti or TiO_2_ NPs in rat or mouse cell lines [31–34]. In a recent study, exposure to microparticles of TiO_2_ resulted in cytotoxic properties and upregulation of pro-inflammatory cytokine gene expression in human macrophages [35]. However, studies on the release of pro-inflammatory cytokines after exposure of human macrophages to NPs have not been conducted. It is possible that mouse cells are more sensitive to exposure to Ti and TiO_2_ NPs, with a more active pro-inflammatory response and greater cytotoxicity than human cells. We previously demonstrated that the pro-inflammatory effect of Ti ions on human macrophages is dependent on the formation of metal–protein aggregates that are phagocytosed by the cells [10]. Differences in the cellular uptake of metal–protein aggregates and particulate Ti or TiO_2_ remain unknown; however, our TEM investigation indicates that there might be a difference. In the present study, and in previous ones conducted, we have shown that Ti metal–protein aggregates are seen inside the cell cytosol, with a clear visible membrane structure (Fig. 7), while no visible membrane is seen around particulate Ti or TiO_2_ (Fig. 7). The cellular mechanism for the uptake and cellular response of soluble and particulate Ti need to be studied further to better understand the different cellular responses. It has been shown that Ti NPs can form bioactive aggregates with calcium and phosphorus, allowing protein binding to the particles. These protein aggregates can then be internalized by the cells. This, in turn, triggers an immune response by secretion of various inflammatory mediators, such as IL-1β, IL-6, tumor necrosis factor (TNF)-α, and prostaglandin E_2_ (PGE2) [36].

It has been proposed that TiO_2_ NPs induce cell damage by oxidative stress, but also cause DNA strand breaks and chromosomal damage [37]. These adverse cell effects are dependent on the form and size of the NPs, as well as their chemical and physical properties. In addition, high doses, time, and exposure route seem to be decisive factors influencing the effects of TiO_2_ NPs on cells [37].

It is known that the release of submicron or Ti NPs into the peri-implant mucosa stimulates the migration of inflammatory cells to the area, which could aggravate the immune response induced by a microorganism [2, 19, 36, 38]. In the present study, we did not observe any effect of micro and NPs on the release of IL-1β from human macrophages; however, the ionic form of Ti induced a strong immunological response. These results indicated that debris from a dental implant into the peri-implant pocket and mucosa might generate a weaker immunological response than that induced by the release of Ti ions from the implant. However, whether these particles are accumulated in the peri-implant mucosa and begin to corrode over time releasing Ti ions, which could, in combination with microbial stimuli, aggravate the immune response and degradation of the supporting tissue remains unknown [2, 4, 17]. Titanium is a very reactive element that needs to be considered as a factor that could be intrinsic to the pathogenesis of peri-implantitis. In the treatment of peri-implantitis, mechanical debridement of the biofilm from the implant surface has been, and still is, the most used, although the success rate of the treatment is relatively low [39]. The extent of particle release from dental implants during mechanical cleaning remains unknown; however, it has been shown that these released particles exert an inflammatory effect, leading to osteolysis of the bone in a mouse model [22]. How these released particles during scaling affect the treatment outcome of peri-implantitis in humans remains unknown but should be taken into consideration. Other treatment methods using an electrochemical method to remove bacterial biofilms from implants have shown promising results but must be investigated further to establish evidence that the method is more efficacious than conventional mechanical debridement [40, 41]. Despite that activation of the inflammasome complex is a key mechanism in degenerative diseases, this is an *in vitro* study that needs to be verified also in clinical studies before the significance for peri-implantitis can be evaluated. To fully explain the mechanism behind Ti-induced inflammasome activation, the expression of involved receptors and mediators needs to be addressed in future.

The main findings in the present study show that Ti ions form metal–protein aggregates that function pro-inflammatory by assembly of NLR3 inflammasome initiating IL-1β release. The present study was conducted at a macrophage cell line, so clinical implication of the results should be with care. Even though several studies have confirmed the pro-inflammatory effect of Ti, little is known about the cellular uptake and the intra cellular mechanism of this process. These cellular processes should be investigated in future studies.

## Conclusion

Ti-induced IL-1β release by macrophages involves activation of the NLRP3 inflammasome. IL-1β release induced by Ti ions activates the NLRP3 inflammasome in contrary to particulate forms of Ti and TiO_2_. Cell death after exposure to Ti ions is linked to the activation of the NLRP3 inflammasome.

## Data Availability

The data used to support the findings of this study are available from the corresponding author upon request.

## References

[1] Weir A, Westerhoff P, Fabricius L, Hristovski K, von Goetz N (2012). Titanium dioxide nanoparticles in food and personal care products. Environmental Science and Technology.

[2] Pettersson M, Kelk P, Belibasakis GN, Bylund D, Molin Thoren M, Johansson A (2017). Titanium ions form particles that activate and execute interleukin-1beta release from lipopolysaccharide-primed macrophages. Journal of periodontal research..

[3] Mangan MSJ, Olhava EJ, Roush WR, Seidel HM, Glick GD, Latz E (2018). Targeting the NLRP3 inflammasome in inflammatory diseases. Nature Reviews. Drug Discovery.

[4] Li X, Tang L, Ye Myat T, Chen D (2020). Titanium ions play a synergistic role in the activation of NLRP3 inflammasome in Jurkat T cells. Inflammation.

[5] Swanson KV, Deng M, Ting JP (2019). The NLRP3 inflammasome: Molecular activation and regulation to therapeutics. Nature reviews Immunology..

[6] Bauernfeind FG, Horvath G, Stutz A, Alnemri ES, MacDonald K, Speert D (2009). Cutting edge: NF-kappaB activating pattern recognition and cytokine receptors license NLRP3 inflammasome activation by regulating NLRP3 expression. Journal of immunology..

[7] Baron L, Gombault A, Fanny M, Villeret B, Savigny F, Guillou N (2015). The NLRP3 inflammasome is activated by nanoparticles through ATP, ADP and adenosine. Cell Death & Disease.

[8] Dostert C, Petrilli V, Van Bruggen R, Steele C, Mossman BT, Tschopp J (2008). Innate immune activation through Nalp3 inflammasome sensing of asbestos and silica. Science.

[9] Moon C, Park HJ, Choi YH, Park EM, Castranova V, Kang JL (2010). Pulmonary inflammation after intraperitoneal administration of ultrafine titanium dioxide (TiO2) at rest or in lungs primed with lipopolysaccharide. Journal of toxicology and environmental health Part A..

[10] Pettersson M, Pettersson J, Molin Thoren M, Johansson A (2018). Effect of cobalt ions on the interaction between macrophages and titanium. Journal of biomedical materials research Part A..

[11] Jo EK, Kim JK, Shin DM, Sasakawa C (2016). Molecular mechanisms regulating NLRP3 inflammasome activation. Cellular & Molecular Immunology.

[12] Chevriaux A, Pilot T, Derangere V, Simonin H, Martine P, Chalmin F (2020). Cathepsin B is required for NLRP3 inflammasome activation in macrophages, through NLRP3 interaction. Front Cell Dev Biol..

[13] Weber K, Schilling JD (2014). Lysosomes integrate metabolic-inflammatory cross-talk in primary macrophage inflammasome activation. The Journal of biological chemistry..

[14] He Y, Hara H, Nunez G (2016). Mechanism and regulation of NLRP3 inflammasome activation. Trends in Biochemical Sciences.

[15] Beigi RD, Kertesy SB, Aquilina G, Dubyak GR (2003). Oxidized ATP (oATP) attenuates proinflammatory signaling via P2 receptor-independent mechanisms. British Journal of Pharmacology.

[16] Garlanda C, Dinarello CA, Mantovani A (2013). The interleukin-1 family: Back to the future. Immunity.

[17] Pettersson M, Pettersson J, Johansson A, Molin Thoren M (2019). Titanium release in peri-implantitis. Journal of Oral Rehabilitation.

[18] Soler, M.D., S.M. Hsu, C. Fares, F. Ren, R.J. Jenkins, L. Gonzaga, et al. 2020. Titanium corrosion in peri-implantitis. *Materials (Basel).* 13(23). 10.3390/ma13235488 10.3390/ma13235488 PMC7730765 33276474

[19] Berryman Z, Bridger L, Hussaini HM, Rich AM, Atieh M, Tawse-Smith A (2020). Titanium particles: An emerging risk factor for peri-implant bone loss. Saudi Dent J..

[20] Delgado-Ruiz, R., and G. Romanos. 2018. Potential causes of titanium particle and ion release in implant dentistry: a systematic review. *International Journal of Molecular Sciences.* 19(11). 10.3390/ijms19113585 10.3390/ijms19113585 PMC6274707 30428596

[21] Wilson TG (2021). Bone loss around implants-is it metallosis?. Journal of periodontology..

[22] Eger M, Sterer N, Liron T, Kohavi D, Gabet Y (2017). Scaling of titanium implants entrains inflammation-induced osteolysis. Science and Reports.

[23] Rasul J, Thakur MK, Maheshwari B, Aga N, Kumar H, Mahajani M (2021). Assessment of titanium level in submucosal plaque around healthy implants and implants with peri-implantitis: A clinical study. Journal of pharmacy & bioallied sciences..

[24] Kelk, P., N.S. Moghbel, J. Hirschfeld, and A. Johansson. 2022. Aggregatibacter actinomycetemcomitans leukotoxin activates the NLRP3 inflammasome and cell-to-cell communication. *Pathogens*. 11(2). 10.3390/pathogens11020159 10.3390/pathogens11020159 PMC8877716 35215102

[25] Repetto G, del Peso A, Zurita JL (2008). Neutral red uptake assay for the estimation of cell viability/cytotoxicity. Nature Protocols.

[26] Spurr AR (1969). A low-viscosity epoxy resin embedding medium for electron microscopy. Journal of Ultrastructure Research.

[27] Schindelin J, Arganda-Carreras I, Frise E, Kaynig V, Longair M, Pietzsch T (2012). Fiji: An open-source platform for biological-image analysis. Nature Methods.

[28] Bergsbaken T, Fink SL, Cookson BT (2009). Pyroptosis: Host cell death and inflammation. Nature Reviews Microbiology.

[29] Tschopp J, Schroder K (2010). NLRP3 inflammasome activation: The convergence of multiple signalling pathways on ROS production?. Nature reviews Immunology..

[30] Di Virgilio F, Dal Ben D, Sarti AC, Giuliani AL, Falzoni S (2017). The P2X7 receptor in infection and inflammation. Immunity.

[31] Hu Q, Zhao F, Fan M, He C, Yang X, Huang Z (2019). The influence of titanium dioxide nanoparticles on their cellular response to macrophage cells. Comparative Biochemistry and Physiology Part C: Toxicology & Pharmacology.

[32] Chen Q, Wang N, Zhu M, Lu J, Zhong H, Xue X (2018). TiO2 nanoparticles cause mitochondrial dysfunction, activate inflammatory responses, and attenuate phagocytosis in macrophages: A proteomic and metabolomic insight. Redox Biology.

[33] Abbasi-Oshaghi E, Mirzaei F, Pourjafar M (2019). NLRP3 inflammasome, oxidative stress, and apoptosis induced in the intestine and liver of rats treated with titanium dioxide nanoparticles: In vivo and in vitro study. International Journal of Nanomedicine.

[34] Zhou Y, Ji J, Ji L, Wang L, Hong F (2019). Respiratory exposure to nano-TiO2 induces pulmonary toxicity in mice involving reactive free radical-activated TGF-beta/Smad/p38MAPK/Wnt pathways. Journal of biomedical materials research Part A..

[35] Ramenzoni, L.L., L.B. Fluckiger, T. Attin, and P.R. Schmidlin. 2021. Effect of titanium and zirconium oxide microparticles on pro-inflammatory response in human macrophages under induced sterile inflammation: an in vitro study. *Materials (Basel).* 14(15). 10.3390/ma14154166 10.3390/ma14154166 PMC8347735 34361359

[36] Messous R, Henriques B, Bousbaa H, Silva FS, Teughels W, Souza JCM (2021). Cytotoxic effects of submicron- and nano-scale titanium debris released from dental implants: An integrative review. Clinical Oral Investigations.

[37] Shabbir, S., M.F. Kulyar, Z.A. Bhutta, P. Boruah, and M. Asif. 2021. Toxicological consequences of titanium dioxide nanoparticles (TiO2NPs) and their jeopardy to human population. *Bionanoscience.* 1–12. 10.1007/s12668-021-00836-3 10.1007/s12668-021-00836-3 PMC7835448 33520589

[38] Charalampakis G, Belibasakis GN (2015). Microbiome of peri-implant infections: Lessons from conventional, molecular and metagenomic analyses. Virulence..

[39] Esposito M, Grusovin MG, Worthington HV (2012). Treatment of peri-implantitis: What interventions are effective? A Cochrane systematic review. European Journal of Oral Implantology.

[40] Schneider S, Rudolph M, Bause V, Terfort A (2018). Electrochemical removal of biofilms from titanium dental implant surfaces. Bioelectrochemistry.

[41] Schlee, M., H.L. Wang, T. Stumpf, U. Brodbeck, D. Bosshardt, and F. Rathe. 2021. Treatment of periimplantitis with electrolytic cleaning versus mechanical and electrolytic cleaning: 18-month results from a randomized controlled clinical trial. *Journal of Clinical Medicine*. 10(16). 10.3390/jcm10163475 10.3390/jcm10163475 PMC8397046 34441770

